# Supplementary material for: Time-series transcriptome analysis identified differentially expressed genes in broiler chicken infected with mixed Eimeria species
Source: Front Genet. 2022 Aug 8;13:886781. doi: 10.3389/fgene.2022.886781 (PMC9393255; doi:10.3389/fgene.2022.886781)
Supplement: Supplementary file 2 [file DataSheet1.ZIP › 7dpi_KEGG.Gsea.1625115961950/KEGG_ALZHEIMERS_DISEASE.html]

Details for gene set KEGG\_ALZHEIMERS\_DISEASE[GSEA]

|  || Dataset | TMM\_7dpi\_gct\_format\_7dpi\_gct\_format.Class\_4dpi.cls #PC\_versus\_NC.Class\_4dpi.cls #PC\_versus\_NC\_repos |
| Phenotype | Class\_4dpi.cls#PC\_versus\_NC\_repos |
| Upregulated in class | 1 |
| GeneSet | KEGG\_ALZHEIMERS\_DISEASE |
| Enrichment Score (ES) | 0.44612497 |
| Normalized Enrichment Score (NES) | 2.2789848 |
| Nominal p-value | 0.0 |
| FDR q-value | 4.1975407E-4 |
| FWER p-Value | 0.0014 |
Table: GSEA Results Summary

  

Fig 1: Enrichment plot: KEGG\_ALZHEIMERS\_DISEASE      
 Profile of the Running ES Score & Positions of GeneSet Members on the Rank Ordered List

  

| SYMBOL | TITLE | RANK IN GENE LIST | RANK METRIC SCORE | RUNNING ES | CORE ENRICHMENT || 1 | COX5A | na | 300 | 0.942 | -0.0076 | Yes |
| 2 | NDUFA2 | na | 303 | 0.935 | 0.0097 | Yes |
| 3 | NDUFB6 | na | 307 | 0.933 | 0.0270 | Yes |
| 4 | NDUFS5 | na | 308 | 0.932 | 0.0444 | Yes |
| 5 | NDUFB7 | na | 323 | 0.915 | 0.0604 | Yes |
| 6 | NDUFB2 | na | 348 | 0.885 | 0.0749 | Yes |
| 7 | NDUFAB1 | na | 356 | 0.876 | 0.0907 | Yes |
| 8 | NDUFA7 | na | 371 | 0.857 | 0.1056 | Yes |
| 9 | NDUFC2 | na | 394 | 0.834 | 0.1193 | Yes |
| 10 | UQCR10 | na | 444 | 0.785 | 0.1299 | Yes |
| 11 | BACE2 | na | 466 | 0.771 | 0.1426 | Yes |
| 12 | CYCS | na | 475 | 0.763 | 0.1562 | Yes |
| 13 | COX6A1 | na | 480 | 0.760 | 0.1701 | Yes |
| 14 | UQCR11 | na | 485 | 0.754 | 0.1839 | Yes |
| 15 | UQCRB | na | 487 | 0.753 | 0.1979 | Yes |
| 16 | COX7A2 | na | 499 | 0.743 | 0.2108 | Yes |
| 17 | CASP8 | na | 514 | 0.732 | 0.2234 | Yes |
| 18 | APBB1 | na | 618 | 0.677 | 0.2274 | Yes |
| 19 | NDUFS3 | na | 623 | 0.675 | 0.2397 | Yes |
| 20 | NDUFB3 | na | 656 | 0.657 | 0.2493 | Yes |
| 21 | NDUFS8 | na | 661 | 0.653 | 0.2612 | Yes |
| 22 | NDUFS6 | na | 686 | 0.642 | 0.2712 | Yes |
| 23 | SDHD | na | 708 | 0.632 | 0.2812 | Yes |
| 24 | NDUFA1 | na | 742 | 0.616 | 0.2900 | Yes |
| 25 | UQCRHL | na | 788 | 0.594 | 0.2973 | Yes |
| 26 | NDUFA5 | na | 794 | 0.593 | 0.3080 | Yes |
| 27 | SDHB | na | 840 | 0.575 | 0.3150 | Yes |
| 28 | NDUFB5 | na | 902 | 0.554 | 0.3202 | Yes |
| 29 | COX6C | na | 930 | 0.546 | 0.3282 | Yes |
| 30 | NDUFV3 | na | 988 | 0.527 | 0.3333 | Yes |
| 31 | NDUFB4 | na | 1015 | 0.517 | 0.3407 | Yes |
| 32 | COX7B | na | 1055 | 0.501 | 0.3468 | Yes |
| 33 | NDUFA6 | na | 1079 | 0.492 | 0.3541 | Yes |
| 34 | NDUFA8 | na | 1080 | 0.492 | 0.3633 | Yes |
| 35 | UQCRQ | na | 1103 | 0.484 | 0.3705 | Yes |
| 36 | NDUFS2 | na | 1124 | 0.477 | 0.3778 | Yes |
| 37 | COX4I1 | na | 1125 | 0.476 | 0.3867 | Yes |
| 38 | SDHC | na | 1135 | 0.473 | 0.3948 | Yes |
| 39 | NDUFB10 | na | 1215 | 0.450 | 0.3966 | Yes |
| 40 | IL1B | na | 1237 | 0.445 | 0.4032 | Yes |
| 41 | UQCRFS1 | na | 1263 | 0.437 | 0.4092 | Yes |
| 42 | ATP5PB | na | 1267 | 0.436 | 0.4171 | Yes |
| 43 | CHP1 | na | 1292 | 0.430 | 0.4232 | Yes |
| 44 | NDUFV1 | na | 1307 | 0.428 | 0.4300 | Yes |
| 45 | NDUFB8 | na | 1330 | 0.423 | 0.4361 | Yes |
| 46 | NDUFS7 | na | 1371 | 0.412 | 0.4404 | Yes |
| 47 | NDUFV2 | na | 1395 | 0.408 | 0.4461 | Yes |
| 48 | BID | na | 1694 | 0.334 | 0.4273 | No |
| 49 | UQCRC2 | na | 1700 | 0.332 | 0.4331 | No |
| 50 | COX7A2L | na | 1761 | 0.316 | 0.4340 | No |
| 51 | NDUFB9 | na | 1842 | 0.299 | 0.4329 | No |
| 52 | CDK5 | na | 1953 | 0.278 | 0.4288 | No |
| 53 | NDUFB1 | na | 1995 | 0.272 | 0.4305 | No |
| 54 | CASP9 | na | 2022 | 0.268 | 0.4333 | No |
| 55 | PLCB4 | na | 2151 | 0.245 | 0.4271 | No |
| 56 | GAPDH | na | 2178 | 0.240 | 0.4295 | No |
| 57 | UQCRC1 | na | 2326 | 0.217 | 0.4212 | No |
| 58 | HSD17B10 | na | 2339 | 0.216 | 0.4242 | No |
| 59 | NDUFA4 | na | 2726 | 0.160 | 0.3948 | No |
| 60 | NDUFA9 | na | 2879 | 0.141 | 0.3846 | No |
| 61 | CAPN2 | na | 3045 | 0.121 | 0.3730 | No |
| 62 | PPP3R1 | na | 3345 | 0.081 | 0.3494 | No |
| 63 | NDUFA10 | na | 3412 | 0.073 | 0.3452 | No |
| 64 | SDHA | na | 3488 | 0.064 | 0.3401 | No |
| 65 | ADAM17 | na | 3490 | 0.064 | 0.3412 | No |
| 66 | CAPN1 | na | 3501 | 0.063 | 0.3415 | No |
| 67 | NDUFS1 | na | 3763 | 0.033 | 0.3202 | No |
| 68 | GNAQ | na | 4006 | 0.004 | 0.3000 | No |
| 69 | APP | na | 4058 | -0.002 | 0.2957 | No |
| 70 | TNFRSF1A | na | 4239 | -0.023 | 0.2810 | No |
| 71 | ATP2A3 | na | 4337 | -0.034 | 0.2735 | No |
| 72 | NDUFS4 | na | 4402 | -0.040 | 0.2689 | No |
| 73 | PPP3CA | na | 4410 | -0.040 | 0.2691 | No |
| 74 | COX7C | na | 4881 | -0.090 | 0.2313 | No |
| 75 | ITPR3 | na | 4900 | -0.092 | 0.2315 | No |
| 76 | SNCA | na | 5117 | -0.114 | 0.2155 | No |
| 77 | CDK5R1 | na | 5261 | -0.127 | 0.2058 | No |
| 78 | CACNA1C | na | 5491 | -0.148 | 0.1893 | No |
| 79 | NCSTN | na | 5700 | -0.170 | 0.1750 | No |
| 80 | PSEN1 | na | 5826 | -0.182 | 0.1680 | No |
| 81 | ATP2A2 | na | 6134 | -0.218 | 0.1462 | No |
| 82 | CASP3 | na | 6448 | -0.248 | 0.1246 | No |
| 83 | CASP7 | na | 6615 | -0.267 | 0.1156 | No |
| 84 | MME | na | 6754 | -0.280 | 0.1093 | No |
| 85 | RYR3 | na | 6821 | -0.288 | 0.1091 | No |
| 86 | APH1A | na | 6922 | -0.299 | 0.1063 | No |
| 87 | BACE1 | na | 7198 | -0.330 | 0.0894 | No |
| 88 | NAE1 | na | 7248 | -0.335 | 0.0915 | No |
| 89 | PLCB1 | na | 7317 | -0.343 | 0.0922 | No |
| 90 | ADAM10 | na | 7357 | -0.347 | 0.0955 | No |
| 91 | CALM2 | na | 7385 | -0.350 | 0.0997 | No |
| 92 | GRIN2C | na | 7410 | -0.352 | 0.1043 | No |
| 93 | ITPR1 | na | 7928 | -0.411 | 0.0686 | No |
| 94 | MAPT | na | 7960 | -0.415 | 0.0737 | No |
| 95 | CALM1 | na | 8103 | -0.435 | 0.0700 | No |
| 96 | EIF2AK3 | na | 8140 | -0.439 | 0.0751 | No |
| 97 | APAF1 | na | 8687 | -0.512 | 0.0388 | No |
| 98 | PPP3CB | na | 8981 | -0.547 | 0.0244 | No |
| 99 | LRP1 | na | 9005 | -0.550 | 0.0328 | No |
| 100 | IDE | na | 9794 | -0.673 | -0.0208 | No |
| 101 | ERN1 | na | 9885 | -0.689 | -0.0155 | No |
| 102 | LPL | na | 10003 | -0.713 | -0.0120 | No |
| 103 | MAPK3 | na | 10320 | -0.774 | -0.0240 | No |
| 104 | PSEN2 | na | 10514 | -0.813 | -0.0250 | No |
| 105 | NOS1 | na | 10720 | -0.858 | -0.0262 | No |
| 106 | PLCB2 | na | 10772 | -0.872 | -0.0142 | No |
| 107 | FADD | na | 10928 | -0.915 | -0.0101 | No |
| 108 | GRIN2A | na | 11237 | -1.012 | -0.0170 | No |
| 109 | ITPR2 | na | 11767 | -1.303 | -0.0370 | No |
| 110 | FAS | na | 11850 | -1.412 | -0.0175 | No |
| 111 | CACNA1D | na | 11943 | -1.649 | 0.0056 | No |
Table: GSEA details [plain text format]

  

Fig 2: KEGG\_ALZHEIMERS\_DISEASE      
 Blue-Pink O' Gram in the Space of the Analyzed GeneSet

  

Fig 3: KEGG\_ALZHEIMERS\_DISEASE: Random ES distribution      
 Gene set null distribution of ES for **KEGG\_ALZHEIMERS\_DISEASE**
